# Ultra-High-Density QTL Marker Mapping for Seedling Photomorphogenesis Mediating Arabidopsis Establishment in Southern Patagonia

**DOI:** 10.3389/fpls.2021.677728

**Published:** 2021-07-23

**Authors:** Daniel Matsusaka, Daniele Filiault, Diego H. Sanchez, Javier F. Botto

**Affiliations:** ^1^IFEVA, UBA, CONICET, Facultad de Agronomía, Universidad de Buenos Aires, Buenos Aires, Argentina; ^2^Gregor Mendel Institute, Austrian Academy of Sciences, Vienna BioCenter, Vienna, Austria

**Keywords:** Arabidopsis, Patagonia, RILs, HIFs, QTL, seedling de-etiolation

## Abstract

*Arabidopsis thaliana* shows a wide range of genetic and trait variation among wild accessions. Because of its unparalleled biological and genomic resources, Arabidopsis has a high potential for the identification of genes underlying ecologically important complex traits, thus providing new insights on genome evolution. Previous research suggested that distinct light responses were crucial for Arabidopsis establishment in a peculiar ecological niche of southern Patagonia. The aim of this study was to explore the genetic basis of contrasting light-associated physiological traits that may have mediated the rapid adaptation to this new environment. From a biparental cross between the photomorphogenic contrasting accessions Patagonia (Pat) and Columbia (Col-0), we generated a novel recombinant inbred line (RIL) population, which was entirely next-generation sequenced to achieve ultra-high-density saturating molecular markers resulting in supreme mapping sensitivity. We validated the quality of the RIL population by quantitative trait loci (QTL) mapping for seedling de-etiolation, finding seven QTLs for hypocotyl length in the dark and continuous blue light (Bc), continuous red light (Rc), and continuous far-red light (FRc). The most relevant QTLs, *Rc1* and *Bc1*, were mapped close together to chromosome V; the former for Rc and Rc/dark, and the latter for Bc, FRc, and dark treatments. The additive effects of both QTLs were confirmed by independent heterogeneous inbred families (HIFs), and we explored *TZP* and *ABA1* as potential candidate genes for *Rc1* and *Bc1*QTLs, respectively. We conclude that the Pat × Col-0 RIL population is a valuable novel genetic resource to explore other adaptive traits in Arabidopsis.

## Introduction

Physical cues of the surrounding environment represent primary agents by which evolution proceeds, imposing selective sieves, which ultimately lead to the buildup of adaptive mechanisms that enable organismal survival. For plants, light, temperature and availability of nutrients play critical roles as a source of abiotic information, controlling a plethora of developmental aspects from germination to flowering (Takou et al., 2019). Quantitative genetics, reverse genetics, and the analysis of natural variation in *Arabidopsis thaliana* (hereafter Arabidopsis) paved the way for the initial identification of genes and alleles variants that are crucial for plant environmental adaptation, regulating key developmental outputs such as skotomorphogenesis, photomorphogenesis, thermomorphogenesis, and innumerable stress-related acclimation responses (Alonso-Blanco et al., 2009; Trontin et al., 2011; Weigel, 2011).

Arabidopsis is a plant model for plant breeding due to its small and simple genome, ample distribution in different environments, and diverse ecological conditions. It is a cosmopolitan species found mostly in the Northern Hemisphere, native to Eurasia and North Africa, although it has been introduced recently to the Americas (Koornneef et al., 2004; Krämer, 2015; Kasulin et al., 2017). Arabidopsis is a common candidate plant for studies on natural genetic variation, because it has a wide range of unequaled genetic resources available (Mitchell-Olds and Schmitt, 2006). Columbia (Col-0) is the reference genome, whereas Patagonia (Pat) is the southernmost accession collected around the world and the first genotype recovered from South America (Kasulin et al., 2017). By whole-genome resequencing and comparative study with 1,135 accessions, we inferred that Pat accession most likely derives from a single homogenous ancestor imported between 100 and 200 generations ago, from Italy or the Balkan/Caucasus regions of Eurasia (Kasulin et al., 2017). Pat was collected in steppes and grasslands from southern Patagonia, where it grows during late spring and summer corresponding to the driest months of the year (∼10 mm per month), when the irradiance is very high (∼2,000 μmol m^2^ s^1^) and the average temperature is around 15°C. The plants conspicuously thrive below Neneo (*Mulinum spinosum*) and Coliguaya (*Colliguaya integerrima*) shrubs, protected from sheep and cow foraging. This suggests that Pat may be adapted to shade as an escape strategy to combined drought, stressful irradiance, and grazing. Supporting this idea, laboratory experiments demonstrated that Pat is hyposensitive to continuous red light (Rc) and shows recalcitrant shade avoidance response in seedlings exposed to far-red (FR) signals simulating neighboring plants (Kasulin et al., 2017). In addition, Pat plants had a strong vernalization requirement associated with high levels of *FLC* expression, typical of high latitude or altitude accessions (Kasulin et al., 2017). The ecology environment of its distribution, its phenotypic characteristics, and the very recent colonization of a narrow Patagonian niche under seemly novel abiotic/biotic interactions, suggest that Pat represents an excellent resource for exploring the genetic and molecular basis of adaptive traits explaining complex developmental responses to light (Kasulin et al., 2017; Legris et al., 2019; Takou et al., 2019).

Seedling photomorphogenesis is a crucial step in the developmental program for the success of young plants. Light induces the transition from skotomorphogenic to the photomorphogenic pattern of plant development in which the transcriptional program is dramatically and finely regulated by HY5, ELONGATED HYPOCOTYL 5, transcription factor (Gangappa and Botto, 2016). The seedling becomes de-etiolated as rapid axis growth ceases, and the cotyledons unfold, expand and become green; whereas the cellular and biochemical components of the photosynthetic machinery are established. De-etiolation is initiated by red (R) and FR light perceived by phytochromes (Quail, 2002), and blue (B) light perceived primarily by cryptochromes (Cashmore et al., 1999). Natural genetic studies found causal photoreceptor genes for seedling de-etiolation (Aukerman et al., 1997; Maloof et al., 2001; Botto et al., 2003; Balasubramanian et al., 2006; Filiault et al., 2008). Furthermore, QTL (quantitative trait loci) mapping found photomorphogenic loci for hypocotyl growth between L*er* and Col-0 (Yanovsky et al., 1997), L*er* and Cvi (Borevitz et al., 2002; Botto et al., 2003), L*er* and No-0 (Magliano et al., 2005), Bay and Sha (Loudet et al., 2008), Col-0 and Kashmir (Wolyn et al., 2004), and advanced intercross RILs between Col-0, Est-1, and Kend-L (Balasubramanian et al., 2009).

In this study, we explored the genetic architecture of seedling photomorphogenic development, aiming at uncovering novel unrecognized causative loci which may eventually help to explain the remarkable invasive capabilities of *A. thaliana* in new regions. For this, we developed a new mapping population from a biparental cross between the reference Col-0 and Pat accessions, which was next-generation-sequenced to increase mapping sensitivity with saturating high-density molecular markers. We validated the quality of the RIL population by QTL mapping for hypocotyl growth in seedlings exposed to different light conditions. Furthermore, we confirmed the most relevant QTLs by a genetic approach through heterogeneous inbred families (HIFs). Empirical data cross-examined with available genomics information suggested that *TANDEM ZINC KNUCLE PROTEIN* (*TZP*) and *ABA DEFICIENT1* (*ABA1*) may be initial candidate genes for *Rc1*a and *Bc1* QTLs in red and blue light signaling, respectively.

## Materials and Methods

### Plant Material

We generated a mapping population of recombinant inbred lines (RILs) derived from parental Col-0 and Pat accessions through single-seed descent hybrids. The Col-0 × Pat RIL population originally comprised 181 independent genotypes amplified until the 6^th^ generation and then next-generation-sequenced. During later reproduction, 19 lines were lost and finally 163 RILs and respective parents were deposited in the Nottingham Arabidopsis Center under stock numbers N2110026 (RILs), N2109973 (Pat), and N2109974 (Col-0). We used null mutants for *TZP* (*tzp-1* and *tzp-2*) in Col-0 background (Zhang et al., 2018) and *ABA1* (*aba1-1*-NW21- and *aba1-6*-CS3772-) in Ler and Col-0 backgrounds, respectively. We included in the experiments the following accessions: Ting-1 (CS76612), Tamm-2 (CS76610), Tamm-27 (CS77341), Sq-1 (CS77266), Got-22 (CS76884), Chat-1 (CS76463), and Ts-1 (CS76615) sharing polymorphisms with *TZP*-Pat; and RRS-10 (CS76592), Ag-0 (CS76430), Pna-17 (CS76575), Dem-4 (CS76794), and Ts-1 (CS76615) sharing polymorphisms with *ABA1*-Pat.

### Experimental Conditions and Design

Seeds were sown in an 0.8% agar medium, stratified in the dark at 5°C for 4 days and further exposed to a 2-h red pulse. After that, seeds were kept 24 h in the dark for homogeneous germination. For the de-etiolation experiments, seedlings were exposed for 5 days under continuous red light (Rc; 30 μmol m^–2^ s^–1^), continuous far-red light (FRc; 0.5 μmol m^–2^ s^–1^), or continuous blue light (Bc; 1 μmol m^–2^ s^–1^), while dark treatment was maintained as control. Light quantity was measured at 660 nm (R), 730 nm (FR), and 470 nm (B) with the spectroradiometer SpectroSense2/2 + meter (Skye Instruments Ltd., Powys, United Kingdom). At the end of the experiments, we photographed seedlings to measure the hypocotyl length with the ImageJ software (Schneider et al., 2012). The experiment was repeated three independent times: in each, we measured at least 10 tallest seedlings per RIL and treatments, whereas control parental lines were replicated with at least 40 seedlings. Data were statistically analyzed by ANOVA.

### DNA Sequencing and Genetic Map

For constructing a genetic map, RIL population and parental lines were low depth short-read next-generation-sequenced. DNA extraction was performed using the standard CTAB method (Doyle and Doyle, 1987). Next-generation-sequencing libraries were prepared according to the Tn5 transposase protocol (Reznikoff, 2008), multiplexing many samples in a single lane sequenced on a HiSeq2500 (Illumina, San Diego, CA, United States) platform. We sequenced the libraries for 181 RILs at the 6th generation plus three independent individual libraries for Col-0 and Pat lines. The data were deposited in ArrayExpress^1^ under accession number E-MTAB-10387. Reads were mapped to TAIR10 reference genome using BWA (Li et al., 2009; Li, 2013) with default parameters, while SNP calling was performed following the GATK pipeline (McKenna et al., 2010) as described in Pisupati et al. (2017). The first step for building a genetic map was to recover a set of reliable parental SNP polymorphisms. To call parental variants, we combined the mapping files of three available libraries for each, which were then filtered for homozygous calls. Additionally, we removed SNPs that could potentially represent spurious heterozygous calls, either because they appeared heterozygous in individual parental libraries or because they overlapped annotated transposable elements. Next, these variants were called in the individual RILs. The resulting SNPs were filtered to only include those at population frequencies between 40 and 60% across lines, with the exception of chromosome IV in which these thresholds were initially relaxed (see below). Genotype calls were inferred from variant calls using Hidden-Markov-Model implemented within the HMM package (Himmelmann, 2015), thus predicting the genotype at each polymorphic position. The genotype call was further refined by SNP binning, establishing consecutive 100 Kb windows of physical chromosomal coordinates. The genotype of each window was determined by the majority of SNPs genotypes, ruling out windows with no majority and/or fewer than 10 called variants. Custom-made workflows for these operations are available at https://github.com/filiaultd/Botto.genotyping.

Subsequently, the 100 Kb SNP binned markers presenting more than 25% of missing observations were removed, as well as those with unusual marker frequency distribution, keeping allele occurrences between 35 and 65% across lines. These were used to construct a genetic linkage map per chromosome using online MST software (Wu et al., 2008)^2^, with Kosambi as mapping function, RIL sixth generation as population type, 163 as number of mapping lines, single linkage-group as grouping logarithm-of-the-odds (LOD) criteria, 2 as no mapping size threshold, 25% as no mapping missing threshold, and 5 cM as no mapping distance threshold (Supplementary Data 1). The assembled genetic map presented 920 informative markers (eliminating the whole first ∼49% of chromosome IV because of strong segregation distortion, see below) (Supplementary Data 1). For reporting chromosomal and total genetic distances, MST-inflated values were corrected with the quickEst function from the R/ASMap package (Taylor and Butler, 2017). Given the unusual marker frequency distribution on chromosome IV, we validated the correct assembly of the mapping population in this area using cleaved-amplified-polymorphic-sequence (CAPS) markers (Konieczny and Ausubel, 1993). Primers were generated at different positions to confirm the expected genotype in consecutive RIL generations, evaluated through positive PCR amplification and Col-0/Pat allele confirmation by digestion with specific restriction enzymes (Supplementary Table 1). PCR conditions were 45 cycles of 30 s, 94°C denaturation; 30 s, 72°C annealing; and 45 s, 72°C extension.

Pat allelic variants for gene candidate assessment were recognized using previous deep next-generation-sequencing data (Kasulin et al., 2017), functionally annotating polymorphisms with SnpEff (Cingolani et al., 2012).

### Quantitative Trait Loci Mapping

Statistics of phenotypic data (Supplementary Data 2) were analyzed with the InfoStat software (Di Rienzo et al., 2001). We calculated the ANOVA mean square (MS), the mean square of the genotype (MSG, among RIL line variance component for each trait), the mean square residual (MSE, residual-error-variance component), the genotypic variance (GV, MSG minus mean square of the residual that is divided for the number of repetitions), the phenotypic variance (PV, MSG divided for the number of repetitions), and the broad-sense heritability (H_2_; average of genetic variance divided by PV).

To perform data mapping, we initially applied Box Cox of MASS library transformation to turn phenotypic non-normal residuals into a normal distribution and homoscedasticity (Venables and Ripley, 2002). With the lme4 package (Bates et al., 2015), we extracted the fitted values from the best model calculating the best-linear-unbiased-estimates (BLUEs) and best-linear-unbiased-predictions (BLUPs). These were used for QTL mapping, conducted with the R/qtl package (Arends et al., 2010). We removed non-informative markers with the drop.nullmarkers function, used the est.rf function to estimate pairwise recombination fractions between all marker pairs and plot them, and applied the calc.genoprob function to calculate conditional genotype probabilities given the multipoint marker data with possible allowance for genotyping errors. Subsequently, composite-interval mapping (CIM) with “hk” method (Haley and Knott, 1992) was applied to calculate the genetic interval coordinates and the LOD score. LOD was considered significant when α < 0.1 after 10,000 permutations performed with the n.perm function, while the % of variability explained (Var) was retrieved fitting a multiple-QTL model using the fitqtl function (Arends et al., 2010). QTLs were named after the most relevant light treatment regarding LOD or % variability explained.

### Confirmation of QTLs and Candidate Gene Exploration

To confirm the presence and the allelic effects on *Rc1* and *Bc1* QTLs, We developed HIFs from individual RILs still segregating to confirm the presence and the allelic effects on Rc1 and Bc1 QTLs (Tuinstra et al., 1997; Laserna et al., 2008). In short, from the progeny of selfed sixth RILs generation presenting heterozygous areas spanning those QTLs of interest (inferred from RIL’s next-generation sequencing), we selected RIL88 and RIL136 for *Rc1* QTL, and RIL20 and RIL71 for *Bc1* QTL. We used CAPS molecular markers to genotype inside and outside the QTL intervals, recovering the informative homozygous seventh generation. PCR conditions were as before, while primers are available in Supplementary Table 5. HIFs were evaluated in de-etiolation experiments along with Col-0 and Pat as controls, following the experimental protocols previously described.

To select candidate genes into the *Rc1* and *Bc1* QTL intervals, we first recognized light-related genes within each QTL interval using manually curated published reports and Gene Ontology Annotations^3^. Having no *a priori* evidence that variation is based on differential expression or structural, we focused on analyzing Pat SNPs of each gene, clustering polymorphisms according to SnpEff high functional impact categories such as missense variant, splice donor variant, splice acceptor variant, and start lost, stop gained, and stop lost codon (Cingolani et al., 2012).

## Results

### Col-0 and Pat Phenotypes in Seedling De-Etiolation

**FIGURE 1:**
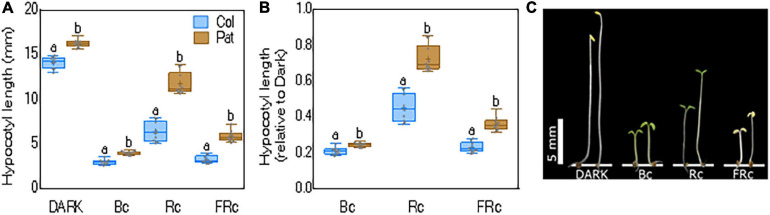
Hypocotyl length for seedling de-etiolation in Col-0 and Pat. Hypocotyl length as **(A)** absolute or **(B)** relative to dark for Col-0 and Pat accessions in R, FR, and B continuous light (Rc = 30 μmol m-2 s-1, FRc = 0.5 μmol m-2 s-1, and Bc = 1 μmol m-2 s-1, respectively). Data are shown as means and SE and significant differences between means are indicated with different letters (LSD test, *p*-value < 0.05). **(C)** Photo shows representative seedlings in each light treatment.

Previously, Kasulin et al. (2017) showed that Col-0 and Pat have contrasting phenotypes under different light conditions. Here, we first confirmed that seedling de-etiolation was reduced in Pat compared with Col-0, a light responsive accession (Figure 1). We exposed Col-0 and Pat etiolated seedlings in Rc, FRc, and Bc light for 5 days, keeping controls in the dark. The most contrasting photomorphogenic phenotypes between accessions were observed in Rc (Col-0 = 6.36 ± 0.33 and Pat = 11.75 ± 0.38 mm). In the dark, the hypocotyl length was also significantly different between accessions (Col-0 = 14.22 ± 0.2 and Pat = 16.22 ± 0.13 mm). To standardize the light response between accessions, we calculated the hypocotyl inhibition response relative to dark (i.e., R/dark in Col-0 = 0.45 ± 0.02 and Pat = 0.72 ± 0.02 mm). In addition, we found significant but lower differences for hypocotyl inhibition response in FRc (Col-0 = 0.23 ± 0.01 and Pat = 0.36 ± 0.01 mm) and Bc (Col-0 = 0.2 ± 0.006, Pat = 0.24 ± 0.003 mm) (Figure 1). These results confirm that Pat accession is hyposensitive to light, and this is particularly significant for seedling de-etiolation in Rc (Figure 1 and Kasulin et al., 2017). The contrasting photomorphogenic responses between Col-0 and Pat suggest that both genotypes are excellent parental accessions to generate a RIL mapping population to understand the genetic architecture of growth-related traits in different light environments.

### Construction of a New Col-0 × Pat RIL Population

**FIGURE 2:**
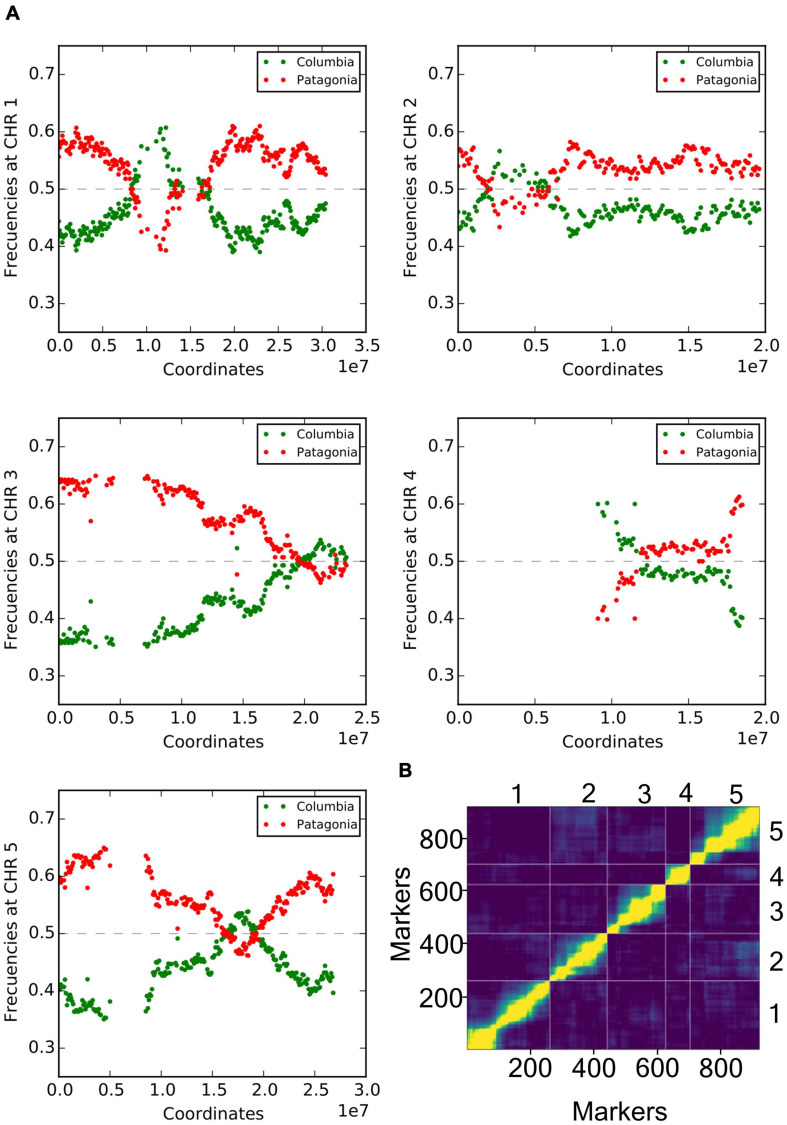
Genetic characterization of Col-0 × Pat RIL population. **(A)** Frequency of allele occurrence for each genotyped marker across the population in each Arabidopsis chromosome. Figures show the distribution of markers as Col-0 or Pat segregation (green and red color, respectively), between 0 (no occurrence) and 1 (100% occurrence in the population). In all chromosomes, the markers ranked for Col-0 or Pat alleles between 35 and 65% occurrence, with the exception for the upper arm of chromosome IV (data not shown). **(B)** Pairwise recombination fractions and LOD scores for all pairs of markers are shown in the upper-left and in the lower-right triangles, respectively, for the five chromosomes. High correlation between markers indicates marker linkage shown in yellow color, while low correlation values indicating unlinked markers is shown in the blue color. The 920 available markers are numerically assigned and placed in physical genetic order, while each chromosome is limited by vertical and horizontal lines.

To analyze the underlying genetic architecture of these observed light-responsive differences, we generated a new RIL population crossing Col-0 × Pat, and F1 descendants were selfed until the sixth generation. These F6 RILs and parental lines were low depth short-read next-generation-sequenced to construct an ultra-high-density genetic map. We used informative polymorphisms for SNP binning, obtaining sequential 100 Kb window genetic markers across the five chromosomes. The genetic call at global population frequency was fitted around 35–65% homozygosity for each parent, while ∼10% were heterozygous (Figure 2A). We found a remarkable frequency distortion in the upper arm of chromosome IV, with a very low representation for Pat markers (Figure 2A). To corroborate that the RIL population had been properly assembled in the early steps of construction, we generated additional CAPS markers in specific positions of the chromosome IV (Supplementary Table 1), and tested F1 hybrid lines and also randomly chosen individuals from ulterior progenies. The results confirmed a correct assemblage, being the F1 strictly heterozygous in all tested positions while posterior generations presented heterozygous or homozygous alleles, as expected (Supplementary Figure 1). We therefore excluded markers corresponding to regions with segregation distortion, and also those presenting missing genotypic data. The genetic map was finally constructed with 920 robust SNP binning markers using the minimum spanning trees algorithm (Wu et al., 2008). Having a corrected total genetic distance of 400.84 cM comparable with previous reports (Supplementary Figure 2 and Supplementary Data 1) (Serin et al., 2017). The order of the markers on the genetic map was used to perform pairwise marker linkage analysis, showing proper correspondence across chromosomes (Figure 2B). The distance between markers was calculated as the total genetic distance divided by the number of markers, amounting to an average of 2.29 cM (Supplementary Table 2).

### Quantitative Trait Loci Mapping for Seedling De-Etiolation

Having developed an appropriate mapping population, we searched QTLs for seedling de-etiolation under Rc, FRc, Bc, and dark conditions (Supplementary Data 2). The RIL population showed wide transgression in all traits. A wide genetic variation in hypocotyl length was observed for RIL seedlings in all traits being most important in dark and Rc (Supplementary Figure 3). In all cases, the broad-sense heritability index was between 0.73 and 0.95, suggesting that a high proportion of phenotypic variation is explained by the genetic component (Supplementary Table 3). These results suggest that these phenotypic data could indeed be informative for QTL mapping to find loci associated with seedling photomorphogenesis.

**FIGURE 3:**
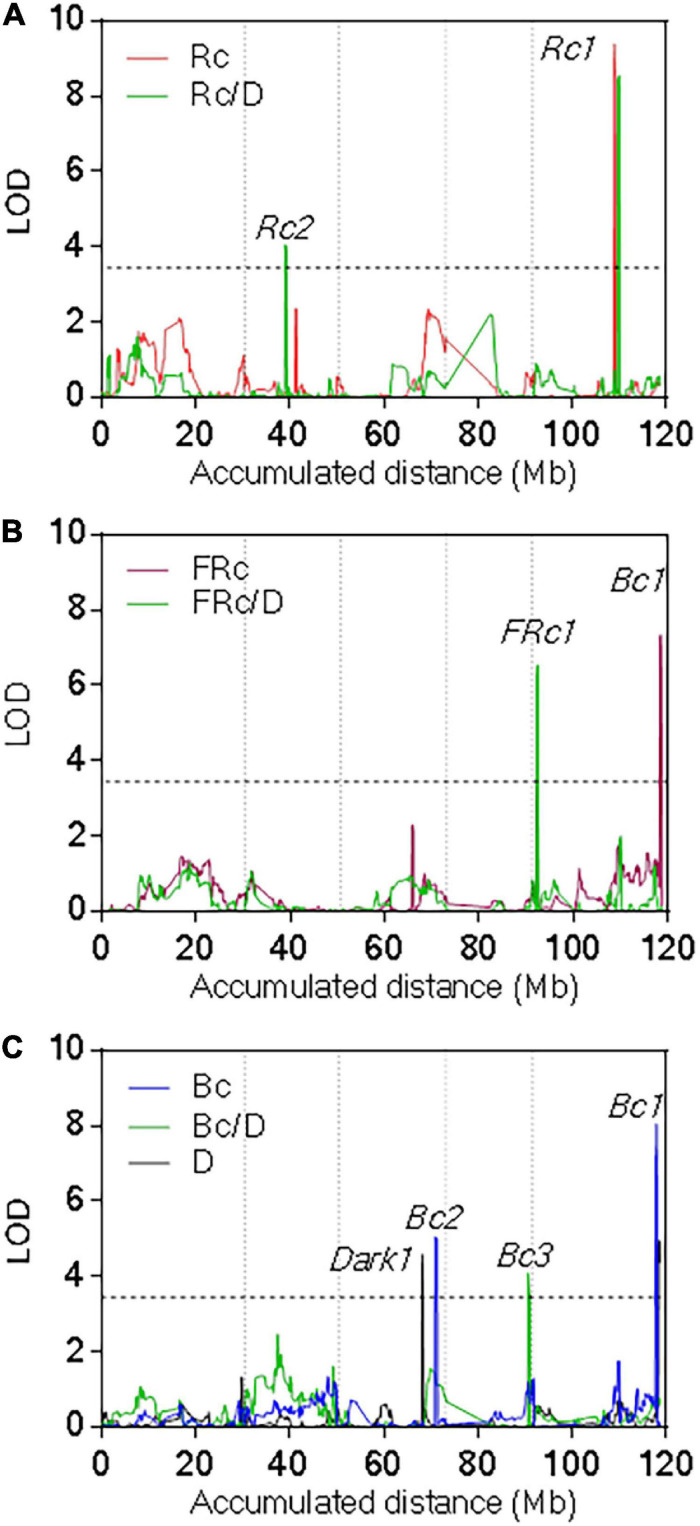
Quantitative trait loci mapping for seedling de-etiolation. QTL mapping for **(A)** Rc and Rc/dark, **(B)** FRc and FRc/dark, and **(C)** Bc, Bc/dark, and dark. LOD score is shown for the accumulated distance of the five chromosomes separated by vertical dashed lines. The horizontal dashed line indicates the significance threshold value.

We found seven QTLs for seedling de-etiolation (Figure 3, Supplementary Figure 4, and Supplementary Table 4). The most significant, *Rc1*QTL, was mapped for Rc and Rc/dark in the middle of the chromosome V (LOD = 9.4, Var = 22%); and *Bc1* QTL was mapped for Bc, FRc, and dark at the end of the same chromosome (LOD = 8, Var = 16%). In both QTLs, the Pat alleles increased the hypocotyl length. *FRc1* QTL was mapped at the top of chromosome V for hypocotyl inhibition in FRc, increasing the Col-0 allele response (LOD = 6.5, Var = 16%). In addition, *Bc2* QTL and *Dark1* QTL were mapped in chromosome III, for Bc and dark, respectively, *Bc3* QTL was mapped for Bc/dark in chromosome IV, and *Rc2* QTL was found for Rc/dark in the middle of chromosome II (Supplementary Figure 4 and Supplementary Table 4).

### Confirmation of Major QTLs by Independent HIFs

**FIGURE 4:**
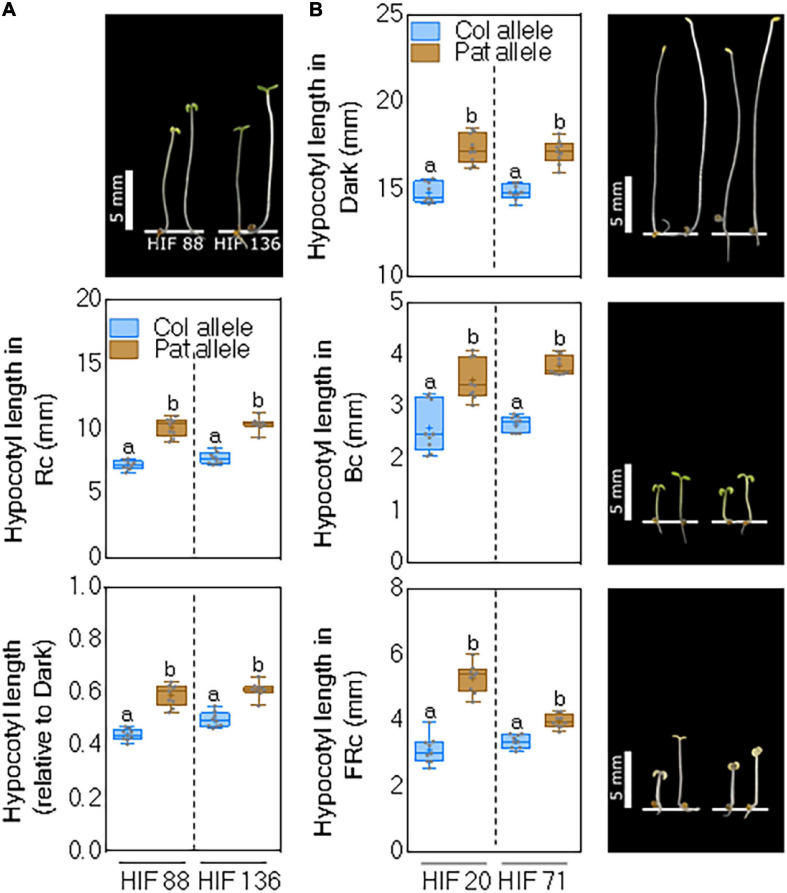
*Rc1* and *Bc1* QTL confirmation by two independent HIFs. **(A)** Hypocotyl length for seedlings grown in Rc and Rc/dark of two independent HIF88 and HIF136 segregating as Col-0 and Pat alleles at the *Rc1* QTL. **(B)** Hypocotyl length for seedlings grown in dark, Bc, and FRc in two independent HIF20 and HIF71 segregating as Col-0 and Pat alleles at the *Bc1* QTL. Box and whisker diagrams depict data around the median, while distinct letters indicate significant differences between means analyzed by LSD test (*p*-value < 0.05). Photos show representative seedlings in each light trait for HIFs segregating as Col-0 (left) and Pat (right).

Since *Rc1* and *Bc1* QTLs were identified as the most significant, we constructed two independent HIFs, segregating on the QTL intervals of interest. For *Rc1* QTL, we used RIL88 and RIL136, displaying heterozygosity within the confidence interval of the QTL, to select homozygous lines segregating between specific CAPS markers (Supplementary Table 5) in the following generation to obtain two independent HIFs. We cultivated the seedlings of both HIFs and evaluated their hypocotyl length in Rc and Rc/dark to confirm the positive effects of Pat alleles. The average response was 6.8 ± 0.13 and 7.4 ± 0.1 mm for the HIF88 and HIF136 carrying Col-0 alleles, and 10.7 ± 0.19 and 10.1 ± 0.22 mm for those HIFs carrying Pat alleles (Figure 4A). *Rc1* QTL was also confirmed for the hypocotyl inhibition response in Rc/dark (Figure 4A). The experimental set was completed assaying HIF88 and HIF136 under Bc and FRc conditions, although these treatments were not mapped for *Rc1* QTL. Bc elicited no consistent differential phenotype, whilst FRc evoked a consistent response for the Pat allele in both HIFs toward increased hypocotyl length (Supplementary Figure 5), overall suggesting this area holds a major light-related gene.

The same experimental approach was carried out to confirm the additive effects of Pat alleles at *Bc1* QTL for seedling de-etiolation, taking advantage of RIL20 and RIL71 with heterozygosity within this QTL area. As expected, HIF20 and HIF71 carrying Pat alleles for *Bc1* QTL increased significantly the hypocotyl length of seedlings grown in the dark, Bc, and FRc compared with those lines carrying Col-0 alleles (Figure 4B). Rc was assayed to complete the set of experiments but provided no further informative phenotype (Supplementary Figure 5).

### Exploration of Potential Causative Genes

The re-sequencing of the new RIL population enabled the unambiguous physical anchoring of ultra-high-density molecular markers, resulting in shorter genetically associated intervals bearing in principle a reduced set of potentially causative genes. Initially, we documented that QTLs presented in the range of 131–458 annotated Col-0 genes (Supplementary Table 6). Subsequently, a variant-call scrutiny of deep sequenced Pat genome compared with Col-0 (Kasulin et al., 2017) allowed the recognition of potentially functional significant Pat alleles, and we documented that 1,006 transcripts (800 annotated coding features) in all QTLs presented high impactful Pat SNPs such as missense, splice donor or splice acceptor variants, start and stop lost codons, or stop codon gained (Supplementary Table 7). We then searched and retrieved light-relevant genes within QTLs from manually curated published reports and GO term analysis, recognizing 30 annotated coding features (Supplementary Table 8; Bae and Choi, 2008; Stamm and Kumar, 2010; Chen and Chory, 2011; Casal, 2013; Xu et al., 2015; Carriedo et al., 2016; Fraser et al., 2016; Gangappa and Botto, 2016; Wang et al., 2020). Cross examination of this information finally revealed that only between one and five genes within each QTL interval exhibited documented roles in light responses and at the same time impactful Pat alleles. All QTLs presented at least one with the exception of *Dark1* QTL (Supplementary Table 9). However, in the following, we restricted the analysis to the most significant *Rc1* and *Bc1* QTLs, confirmed through two independent HIFs (Figure 4).

**FIGURE 5:**
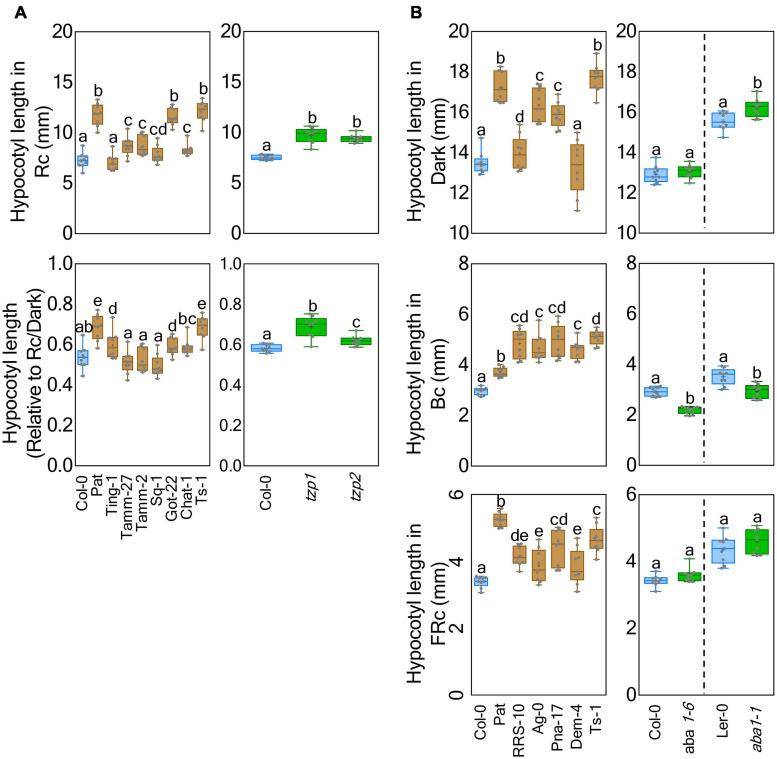
*TZP1* and *ABA1* as candidate genes for *Rc1* and *Bc1* QTLs, respectively. **(A)** Seedling hypocotyl length was measured under Rc and Rc/dark conditions for accessions Ting-1 (CS76612), Tamm-2 (CS76610), Tamm-27 (CS77341), Sq-1 (CS77266), Got-22 (CS76884), Chat-1 (CS76463), and Ts-1 (CS76615) that shared some or all *TZP-*Pat polymorphisms; and in parallel *tzp-1* and *tzp-2* with corresponding Col-0 as a control. **(B)** Seedling hypocotyl length was measured under dark, Bc, and FRc conditions for accessions RRS-10 (CS76592), Ag-0 (CS76430), Pna-17 (CS76575), Dem-4 (CS76794), and Ts-1 (CS76615) that shared all *ABA1*-Pat polymorphisms; and in parallel *aba1-1* and *aba1-6* with the corresponding Ler and Col-0 controls, respectively. Box and whisker diagrams depict data around the median, while distinct letters indicate significant differences between means analyzed by LSD test (*p*-value < 0.05).

Light-related *ABCI21*, *FLS6*, *RPP8*, *TZP*, and *YUC5* genes displayed several informative Pat polymorphisms within *Rc1* QTL interval (Supplementary Table 9). We focused on *TZP*, since it has been implicated in de-etiolation and photoperiodic responses (Loudet et al., 2008; Kaiserli et al., 2015; Zhang et al., 2018), and *TZP*-Pat presented a high number of missense variants (#6), with an additional stop codon gained at the end portion of the protein (Supplementary Table 10). Then, we analyzed under Rc and Rc/dark six accessions sharing half *TZP*-Pat missense variants (Ting-1, Tamm-2, Tamm-27, Sq-1, Got-22, and Chat-1), and one extra accession sharing all of them (Ts-1) (Supplementary Table 11). The former accessions displayed a light response more similar to Col-0, but Ts-1 was hyposensitive to light in the same way as Pat (Figure 5A). We also found that seedlings of two *tzp* loss-of-function were longer than Col-0 in Rc, Rc/dark and also FRc, but not in Bc; this is comparable with the Pat contribution in *Rc1* QTL with the exception of the Bc response (Figure 5A and Supplementary Figure 6). These results, together with the HIF phenotypes, may suggest that Pat carries a weak *TZP* allele with its contribution in phytochrome but not cryptochrome signaling.

On the other hand, only *RPT3, ABA1*, and a kinectin-like protein (*AT5G66250)* presented informative Pat polymorphisms for *Bc1*QTL (Supplementary Table 9). We also followed up the analysis of *ABA1* as the more impactful gene bearing five missense variants (Supplementary Table 10), because it has been previously associated with skotomorphogenesis (Barrero et al., 2008). Using the same previous experimental approach, we assayed five accessions sharing all *ABA1*-Pat polymorphisms (RRS-10, Ag-0, Pna-17, Dem-4, and Ts-1) (Supplementary Table 11). These showed longer hypocotyls in Bc and FRc than Col-0, whereas under dark conditions only Dem-4 accession did not have a response similar to that of Pat (Figure 5B). We also studied the phenotype of *aba1-1* and *aba1-6* mutants compared with L*er* and Col-0, respectively. In Bc light, both *aba1* mutant seedlings were shorter than respective wild-types; while we did not find consistent hypocotyl differences between genotypes in dark, FRc, and Rc (Figure 5B and Supplementary Figure 6). Although accessions sharing *ABA1*-Pat polymorphisms showed overall similar responses to Pat as expected from *Bc1* QTL, the lack of informative dark and FRc *ABA1*-dependent phenotypes in L*er* and Col-0 mutants is inconsistent with the idea that *ABA1* may drive causation. However, we cannot, at present, rule out that *ABA1*-Pat may exert the expected photomorphogenesis regulatory role in the context of Pat epistatic interactions.

## Discussion

Here, we generated a new RIL population between Col-0 reference genome and Pat, a new accession reported in Patagonia as the genotype collected in the Southernmost area of *A. thaliana* distribution (Kasulin et al., 2017). To confirm the quality of the genetic resource developed, we mapped QTLs for seedling de-etiolation. These light responses were previously documented to be contrasting traits between Col-0 and Pat (Kasulin et al., 2017). We found seven QTL for seedling de-etiolation and confirmed the major *Rc1* and *Bc1* QTLs by two independent HIFs. We also explored causative genes, and identified six and three light-related genes into *Rc1* and *Bc1* QTL intervals, respectively, with potential informative polymorphism between Col-0 and Pat. By physiological studies using mutants and accessions sharing Pat polymorphisms, we suggest that *TZP* and perhaps *ABA1* are candidate genes for *Rc1* and *Bc1* QTLs, respectively.

We developed a new re-sequenced 163 RILs between Col-0 and Pat parental lines and constructed a genetic map with 920 informative ultra-high-density markers with an average distance between them of 2.295 cM for the five chromosomes (Supplementary Table 2). Although in most genomic regions we found an expected segregation of markers between 35 and 65% for Col-0 and Pat alleles (Figure 2), it is clear that segregation distortion was obtained for a large portion of chromosome IV (a gap of 9,100,000 Mb). In this genomic area, we found higher segregation of Col-0 alleles compared with Pat alleles. We confirmed the correct assemblage of the population, being the F1 strictly heterozygous alleles in all tested positions, while posterior generations presented heterozygous or homozygous alleles (Supplementary Figure 1). Then, we excluded 273 markers in the upper area of chromosome IV for QTL mapping analysis. Although the segregation distortion area in chromosome IV includes a known characterized paracentric inversion (Fransz et al., 2016), allele biases of the RIL population have been usually documented as a consequence of parental incompatibilities leading to fertility/sterility problems, which increase the allele frequency of one parental line during RIL construction (Alonso-Blanco et al., 1998; Loudet et al., 2002; Törjék et al., 2006).

From the seven QTLs found for seedling de-etiolation, the most significant QTL were mapped in chromosome V (*Rc1* and *Bc1*). The additive effects of *Rc1* and *Bc1* QTLs were confirmed by HIFs. *Rc1* QTL was mapped for hypocotyl growth in Rc and Rc/dark inhibition response and explained 20% of the phenotypic variation and Pat alleles increasing the traits (Supplementary Table 4). We confirmed *Rc1* QTL by two independent HIFs into an interval between 45.9 and 51.8 cM, where 458 genes were mapped (Supplementary Table 6). To select candidate genes into the *Rc1* QTL interval, we analyzed missense variants, splice donor variants, splice acceptor variants, start lost, stop gained, and stop lost codons occurring in genes previously suggested to be involved in light responses, finding five falling into these categories (Supplementary Table 9). *TZP* (*AT5G43630*) in Pat carries six non-synonymous SNPs and an additional C786T SNP that produced a premature stop codon (Supplementary Table 9). Importantly, *LIGHT5* QTL was also mapped for hypocotyl growth in white light in the Bay × Sha RIL population in the same position of *Rc1* QTL (Loudet et al., 2008). *TZP* is the gene responsible for *LIGHT5* QTL whose allele in the Bay accession resulted in shorter hypocotyls in blue and white light (Loudet et al., 2008; Coluccio et al., 2011). We therefore suggest *TZP* as candidate gene for *Rc1* QTL for the following correlations: (i) the accessions sharing the same *TZP*-Pat polymorphisms (at least three of the six missense variants) showed the same seedling hyposensitive phenotype compared with Pat (Got-22 and Ts-1 in Rc and Ts-1 Rc/dark traits); (ii) *tzp-1* and *tzp-2* null mutant seedlings showed longer hypocotyls and lower inhibition response than Col-0, and these results are according to the phenotype of Pat that carries a hyposensitive *TZP* allele; and (iii) *TZP*-Pat also carries a premature stop codon comparable with the reported Bay allele. Interestingly, cryptochromes and phytochromes pathways can act antagonistically in photomorphogenesis (Casal, 2000; Su et al., 2015), and *TZP* seems to play a key role in the integration of these light signaling pathways as evidenced by the contrasting phenotypes of HIF88 and HIF136 and *tzp-1* and *tzp-2* mutant seedlings in Bc vs. Rc and FRc (Figures 4A, 5A and Supplementary Figures 5, 6). Upon exposure to R light, Kaiserli et al. (2015) demonstrated that *TZP* is quickly recruited by the phytochrome B (phyB) to nuclear photobodies in which it acts as a link to mark sites of active transcription. TZP can also interact physically with phyA and phyB to regulate the degradation of *FAR-RED ELONGATED HYPOCOTYL1 (FHYI)*, through protein–protein interaction (Zhang et al., 2018). In addition, TZP not only acts as a positive regulator of phytochrome-mediated responses but also as a negative regulator of blue light in seedling photomorphogenesis (Zhang et al., 2018). We suggest that the *TZP-*Pat allele that carries a stop codon in the PLUS3 domain is less functional and confers R light hyposensitive by affecting protein–protein interactions. Further empirical experimentation introgressing *TZP*-Pat into a contrasting isogenic background will be required to confirm the proposed causation.

*Bc1* QTL was mapped for seedling de-etiolation in FRc, Bc, and dark at the lower arm of chromosome V, and Pat alleles increased the hypocotyl growth (Figure 5 and Supplementary Table 4). *Bc1* QTL exemplifies the difficulty to distinguish between the effect of a single gene pleiotropic to different environments or multiple genes in close linkage contributing in specific environments, as suggested for the partial interval overlapping between FRc (26,200–26,700 kb), Bc (25,700–26,200 kb), and dark (26,300–26,800 kb). Overlapping with *Bc1* QTL, Wolyn et al. (2004) mapped *FR5* QTL for hypocotyl length in FRc in the RIL population between Col-*gl1* and Kas-1. In our case, only three previously recognized light-related genes presented Pat impactful alleles into the *Bc1* QTL interval, and *ABA1 (AT5G67030*) was explored as the candidate gene. *ABA1* encodes a single copy zeaxanthin epoxidase gene involved in the first step of the biosynthesis of abscisic acid (ABA), and *aba1* mutant plants show reduced growth that can be rescued by spraying with an ABA solution (Koornneef et al., 1982, 1998; Leung and Giraudat, 1998). We found that the four of the five accessions sharing *ABA1*-Pat polymorphisms showed hypocotyls longer than Col-0 in dark, Bc, and FRc traits; while *aba1*-1 and *aba1*-6 seedlings were significantly shorter than wild-types in Bc (Figure 5B). The *aba1* mutant response in Bc is in line with previous observations, where it displayed a partial de-etiolated phenotype such as reduced hypocotyl growth, cotyledon expansion, and impaired carotenoid biosynthesis in late skotomorphogenic growth (Barrero et al., 2008). According to this evidence, the results may suggest that *ABA1*-Pat could be more functional than the *ABA1*-Col-0 allele. However, mutated seedlings were not significantly shorter than wild-types in dark and FRc, inconsistent with the expected additive effects of *Bc1* QTL. Therefore, additional experiments will be needed to clarify the role of *ABA1* during de-etiolation and causation in Pat light responses.

Natural variation affecting ecological traits can be analyzed and relatively quickly exploited in Arabidopsis. The novel re-sequenced Col-0 × Pat RIL population is a valuable genetic resource for revealing new genes or allelic variants involved in the signaling developmental program, stress responses, or any future trait of interest for the scientific community. In this study, we demonstrated that Col-0 × Pat is a rich genetic resource to identify the genetic and molecular basis of adaptive traits linked with phenotypic variation and to evaluate ecological pressure clues in Arabidopsis adaptation.

## Data Availability Statement

The datasets presented in this study can be found in online repositories. The names of the repository/repositories and accession number(s) can be found below: Arrayexpress (EMBL-EBI), E-MTAB-10387; NASC, N2110026 (RILs), N2109973 (Pat), and N2109974 (Col-0).

## Author Contributions

JB conceived the project. DF generated the genetic map of the RIL population. DS contributed to the bioinformatics analysis. DM performed the physiological and QTL mapping experiments. DM, DS, and JB interpreted the data and wrote the manuscript. All the authors read and approved the final version of the manuscript.

## Conflict of Interest

The authors declare that the research was conducted in the absence of any commercial or financial relationships that could be construed as a potential conflict of interest.

## Publisher’s Note

All claims expressed in this article are solely those of the authors and do not necessarily represent those of their affiliated organizations, or those of the publisher, the editors and the reviewers. Any product that may be evaluated in this article, or claim that may be made by its manufacturer, is not guaranteed or endorsed by the publisher.

## Footnotes

1 www.ebi.ac.uk/arrayexpress/

2 http://www.mstmap.org/

3 https://www.arabidopsis.org

## Supplementary Material

Supplementary Figure 1 CAPS PCR assessing allele segregation at chromosome IV in four positions. In panel **(A)**, the figure shows parental homocigous alleles at different generations and heterozygosis of the original F1 individual, called in four coordinates of chromosome IV. **(B)** Segregation in random F2 individuals for previous coordinates. Arrows point to the inferred genotypes (red = Pat, blue = Col-0, green = heterozygous). Different restriction enzymes were used: *Bam*HI for P1 and *Eco*RI for P2–P4.

Supplementary Figure 2 **(A)** The chart illustrates genotype data of 920 markers (*x*-axes) into the five chromosomes (divided by vertical lines black) with black points for 163 individuals of population between Col-0 and Pat (*y*-axis). From the 163 RILs, 55% segregated as Pat and 45% segregated as Col-0 (not included heterozygote lines). **(B)** The chart shows marker position in centimogans (cM) in function of the five chromosomes (*x*-axes). The horizontal bars into the chart represent the marker position in the genetic map. In some areas, there are saturated markers in the map.

Supplementary Figure 3 Phenotypic distribution for seedling de-etiolation in 163 RILs between Col-0 and Pat. Hypocotyl length (mm) in Rc, Bc, FRc, and dark. Blue and red arrows indicate the average response for Col-0 and Pat, respectively.

Supplementary Figure 4 Quantitative trait loci positions into the five chromosomes of *Arabidopsis thaliana* for seedling de-etiolation. The genetic distance is indicated in Mb, centromeres (black) and QTLs (green). Markers for confirmation of *Rc1* and *Bc1* QTLs are indicated.

Supplementary Figure 5 *Rc1 and Bc1* QTLs HIFs under come informative light treatment conditions. **(A)** Hypocotyl length in Bc and FRc for HIF88 and HIF136 segregating as Col-0 and Pat alleles at the *Rc1* QTL. **(B)** Hypocotyl length in Rc for HIF20 and HIF71 segregating as Col-0 and Pat alleles at the *Bc1* QTL. In all cases, box and whisker diagrams depict data around the median, while distinct letters indicate significant differences between means analyzed by LSD test (*p*-value < 0.05). Photos show representative seedlings in each light trait for HIFs segregating as Col-0 (left) and Pat (right).

Supplementary Figure 6 Hypocotyl length of mutants under informative light treatment conditions. **(A)** *tzp-1* and *tzp-2* mutants in Bc and FRc. **(B)** *aba1-1* and *aba1-6* mutants in Rc. Box and whisker diagrams depict data around the median, while distinct letters indicate significant differences with the respective wild-type control. Statistical differences between means were analyzed by LSD test (*p*-value < 0.05).

Supplementary Table 1 Cleaved-amplified-polymorphic-sequence primers used for segregation distortion in chromosome IV.

Supplementary Table 2 Per chromosome summary for markers used to construct the genetic map of RIL population between Col-0 and Pat.

Supplementary Table 3 Statistics summary for hypocotyl length in seedling de-etiolation.

Supplementary Table 4 Quantitative trait loci (QTL) for seedling de-etiolation.

Supplementary Table 5 Cleaved-amplified-polymorphic-sequence primers used for *Rc1* and *Bc1* QTL segregation.

Supplementary Table 6 Annotated genes within each QTL interval.

Supplementary Table 7 Inferred impactful Pat alleles occurring within the seven QTLs.

Supplementary Table 8 Light-related genes recognized within the seven QTLs.

Supplementary Table 9 Candidate genes involved in de-etiolation responses occurring within each QTL.

Supplementary Table 10 Pat polymorphisms occurring in genes *TZP* and *ABA1*.

Supplementary Table 11 Accessions with the same informative Pat missense variants in candidate genes *TZP* and *ABA1*.

Supplementary Data 1 Col-0 × Pat RILs genetic map and initial data input per chromosome for MST map construction.

Supplementary Data 2 RIL phenotypic data for light-treatment QTL mapping.
