# Supplementary material for: Ultra-High-Density QTL Marker Mapping for Seedling Photomorphogenesis Mediating Arabidopsis Establishment in Southern Patagonia
Source: Front Plant Sci. 2021 Jul 23;12:677728. doi: 10.3389/fpls.2021.677728 (PMC8343176; doi:10.3389/fpls.2021.677728)
Supplement: Supplementary Figure 1 — CAPS PCR assessing allele segregation at chromosome IV in four positions. In panel (A), the figure shows parental homocigous alleles at different generations and heterozygosis of the original F1 individual, called in four coordinates of chromosome IV. (B) Segregation in random F2 individuals for previous coordinates. Arrows point to the inferred genotypes (red = Pat, blue = Col-0, green = heterozygous). Different restriction enzymes were used: BamHI for P1 and EcoRI for P2–P4. [file Data_Sheet_1.zip › Supplementary Figures 1-6.pdf]

Suppl. Figure 1

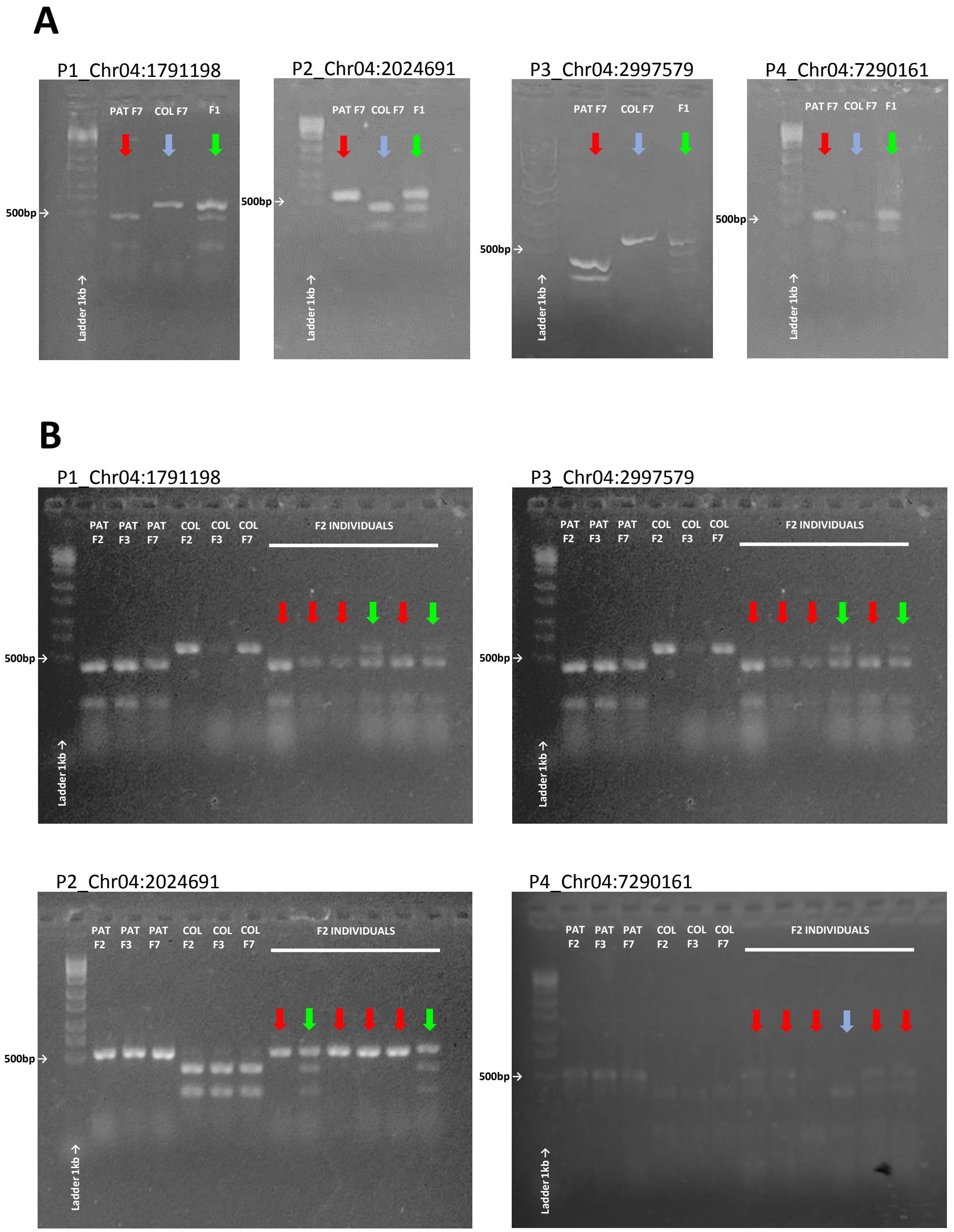

Suppl. Figure 1. CAPS PCR assessing allele segregation at the chromosome IV in four positions. In (A), the figure shows parental homocigous alleles at different generations and heterozygosis of the original F1 individual, called in four coordinates of chromosome IV. In (B), segregation in random F2 individuals for previous coordinates. Arrows point to the inferred genotypes (Red = Pat, Blue = Col-0, Green = heterozygous). Different restriction enzymes were used: BamHI for P1 and EcoRI for P2 to P4.

Suppl. Figure 2

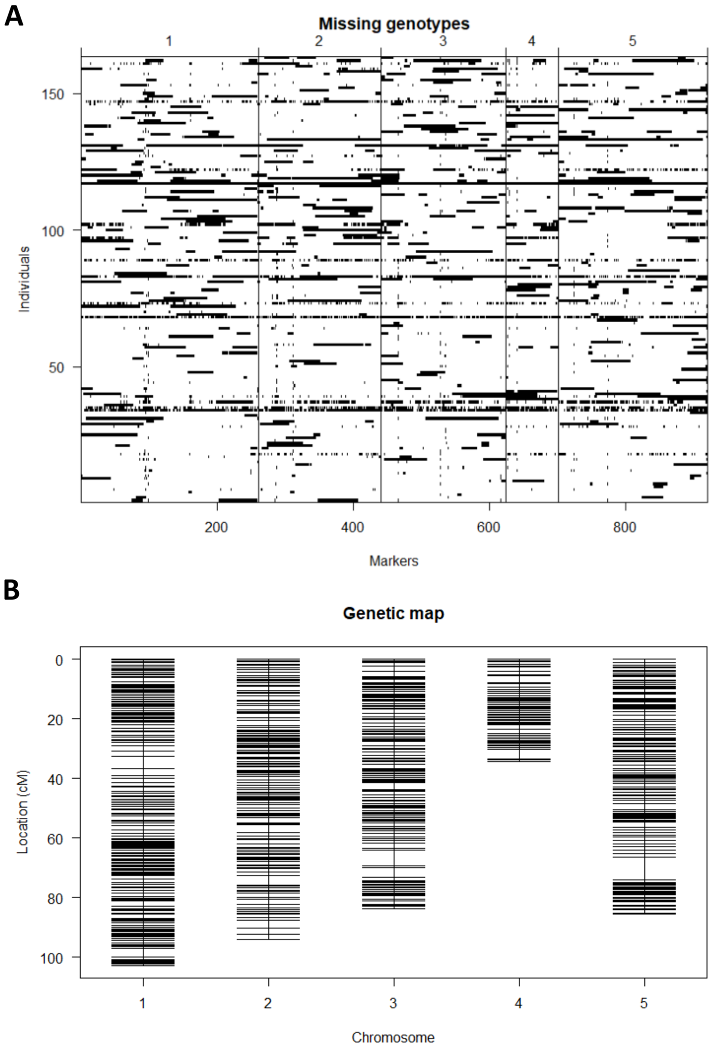

Suppl. Figure 2. (A) The chart illustrates genotype data of 920 markers (x axes) into the five chromosomes (divided by vertical lines black) with black points for 163 individuals of population between Col-0 and Pat (y axis). From the 163 RILs, 55% segregated as Pat and 45% segregated as Col-0 (not included heterozygote lines). (B) The chart shows marker position in centimogans (cM) in function of the five chromosomes (x axes). The horizontal bars into the chart represent the marker position in the genetic map. In some areas, there are saturated markers in the map.

Suppl. Figure 3

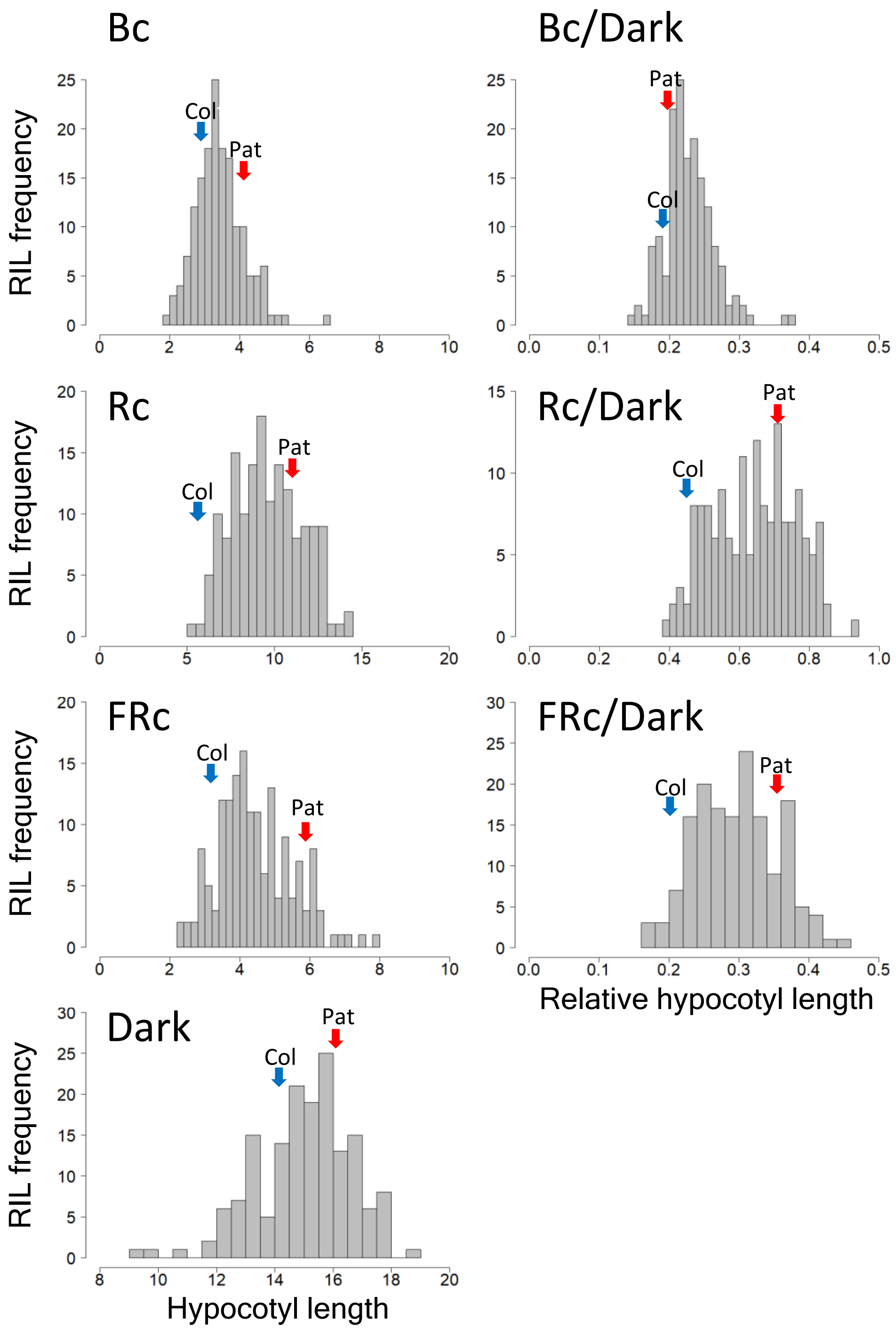

Suppl. Figure 3. Phenotypic distribution for seedling de-etiolation in 163 RILs between Col-0 and Pat. Hypocotyl length (mm) in Bc, Rc, FRc and dark. Blue and red arrows indicate the average response for Col-0 and Pat, respectively.

Suppl. Figure 4

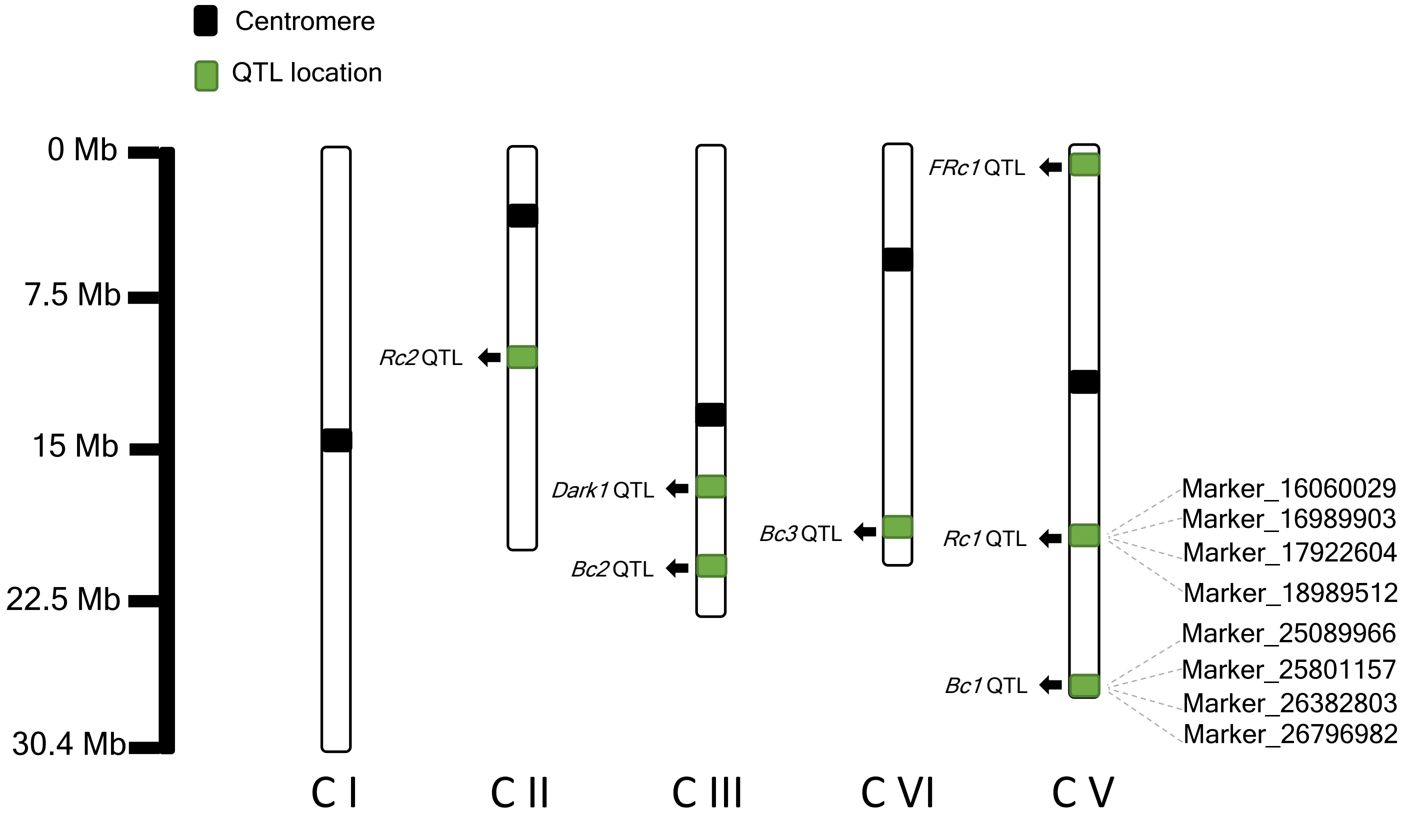

Suppl. Figure 4. QTL positions into the five chromosomes of *A. thaliana* for seedling de-etiolation. The genetic distance is indicated in Mb, centromeres (black) and QTLs (green). Markers for confirmation of *Rc1* and *Bc1* QTLs are indicated.

Suppl. Figure 5

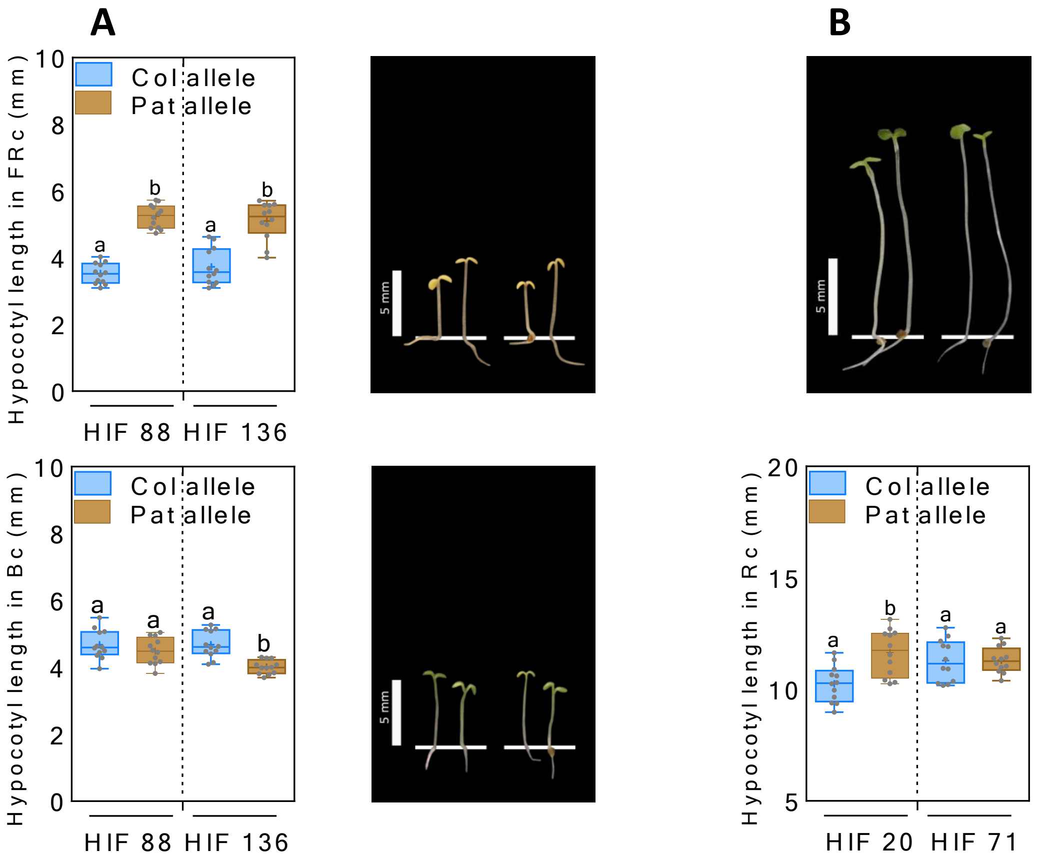

Suppl. Figure 5. *Rc1 and Bc1* QTLs HIFs under informative light treatments. (A) Hypocotyl length in Bc and FRc for HIF88 and HIF136 segregating as Col-0 and Pat alleles at the *Rc1* QTL. (B) Hypocotyl length in Rc for HIF20 and HIF71 segregating as Col-0 and Pat alleles at the *Bc1* QTL. In all cases, box and whisker diagrams depict data around the median, while distinct letters indicate significant differences between means analyzed by LSD test (p-value <0.05). Photos show representative seedlings in each light trait for HIFs segregating as Col-0 (left) and Pat (right).

Suppl. Figure 6

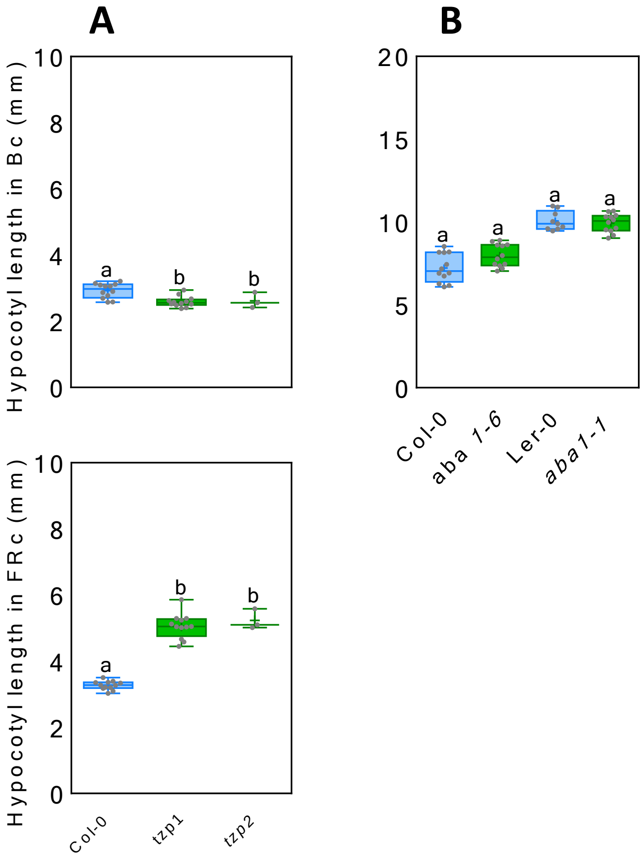

Suppl. Figure 6. Hypocotyl length of mutants under informative light treatments. (A) *tzp-1* and *tzp-2* mutants in Bc and FRc. (B) *aba1-1* and *aba1-6* mutants in Rc. Box and whisker diagrams depict data around the median, while distinct letters indicate significant differences with the respective wild-type control. Statistical differences between means were analyzed by LSD test (p-value <0.05).
