# Supplementary material for: Ultra-High-Density QTL Marker Mapping for Seedling Photomorphogenesis Mediating Arabidopsis Establishment in Southern Patagonia
Source: Front Plant Sci. 2021 Jul 23;12:677728. doi: 10.3389/fpls.2021.677728 (PMC8343176; doi:10.3389/fpls.2021.677728)
Supplement: Supplementary Figure 1 — CAPS PCR assessing allele segregation at chromosome IV in four positions. In panel (A), the figure shows parental homocigous alleles at different generations and heterozygosis of the original F1 individual, called in four coordinates of chromosome IV. (B) Segregation in random F2 individuals for previous coordinates. Arrows point to the inferred genotypes (red = Pat, blue = Col-0, green = heterozygous). Different restriction enzymes were used: BamHI for P1 and EcoRI for P2–P4. [file Data_Sheet_1.zip › Supplementary Tables 1-6 and 9-11.docx]

**Suppl. Table 1: CAPS primers used for segregation distortion in the chromosome IV.**

| ID | Target location in chromosome IV | Restriction  enzymes | Forward primer  (5' to 3') | Reverse primer (5' to 3' on opposite strand) |
| --- | --- | --- | --- | --- |
| P1 | 1791198 | BamHI | GCCCGACCTTCTACAGCT | TCCTGTTTTGCCCTCTCCC |
| P2 | 2024691 | EcoRI | GCCAGCCCTAAACGACGT | CCATCCATGACTGCTCCGT |
| P3 | 2997579 | EcoRI | GCTGTAGAGAGTGGGCTGC | CGTGGAGCTTCTGGGATCTC |
| P4 | 7290161 | EcoRI | ACCACCACTACCAGTACTCT | ACGCAATGCAAAACCCTGG |

**Suppl. Table 2. Per chromosome summary for markers used to construct the genetic map of RIL population between Col-0 and Pat.**

| Chr | Markers | Total length  (cM) | Aver. Distance (cM) | Maximun gap  (cM) | |
| --- | --- | --- | --- | --- | --- |
| chr 1 | 260 | 102.82 | 2.53 | 4.02 | |
| chr 2 | 180 | 94.19 | 1.91 | 2.99 | |
| chr 3 | 184 | 83.85 | 2.19 | 5.47 | |
| chr 4 | 77 | 34.26 | 2.25 | 3.27 | |
| chr 5 | 219 | 85.71 | 2.55 | 7.64 | |
| Total | 920 | 400.84 | 2.29 | | 4.67 |

**Suppl. Table 3. Statistics summary for seedling de-etiolation.**

|  | dark (mm) | Bc (mm) | Bc/dark | Rc (mm) | Rc/dark | FRc (mm) | FRc/dark |
| --- | --- | --- | --- | --- | --- | --- | --- |
| Pat | 16.22±0.13 | 3.98±0.06 | 0.24±0.01 | 11.75±0.38 | 0.72±0.02 | 5.85±0.19 | 0.36±0.01 |
| Col-0 | 14.12±0.20 | 2.95±0.08 | 0.20±0.01 | 6.36±0.33 | 0.45±0.02 | 3.24±0.12 | 0.22±0.01 |
| RIL mean | 15.02±0.12 | 3.42±0.05 | 0.22±0.01 | 9.57±0.15 | 0.64±0.01 | 4.44±0.08 | 0.29±0.01 |
| RIL max-min | 18.94-9.32 | 6.45-1.91 | 0.37-0.14 | 14.07-5.11 | 0.92-0.41 | 7.8-2.23 | 0.44-0.16 |
| CMG | 7.99 | 1.38 | 0.0037 | 11.42 | 0.4 | 3.36 | 0.1 |
| CME | 1.58 | 0.21 | 0.00093 | 2.76 | 0.1 | 0.9 | 0.004 |
| VG | 2.136 | 0.39 | 0.00092 | 2.886 | 0.1 | 0.82 | 0.031 |
| VF | 2.663 | 0.46 | 0.00123 | 3.806 | 0.133 | 1.12 | 0.033 |
| H_2_ | 0.802 | 0.847 | 0.748 | 0.758 | 0.75 | 0.73 | 0.953 |

CMG is the variance component for each trait among RIL lines.

CME is the residual (error) variance component for each trait among RIL lines.

VG is the genotypic variance for each trait among RIL lines.

VF is the phenotypic variance for each trait among RIL lines.

H_2_ is defined as herability that is the measure of VG/VF.

**Suppl. Table 4. Quantitative trait loci (QTL) for seedling de-etiolation.**

QTL for hypocotyl length under Rc, FRc, Bc, dark, Rc/dark, FRc/dark and Bc/dark traits. The table shows significant QTL (α = 0.10). For each QTL, it is indicated the chromosome number, trait, map position (cM), 2-LOD interval in physical distance (bp), LOD, % variability explained and additive effects (the positive and negative signs indicate Col-0 alleles and Pat alleles increase the average response of the trait, respectively). The number of QTL is arbitrary in order to the LOD value.

| Chr | QTL | Trait | Map pos (cM) | Interval (cM) | Interval (Kb) | | LOD | % Var | | Additive | | |
| --- | --- | --- | --- | --- | --- | --- | --- | --- | --- | --- | --- | --- |
| 5 | *Rc1* | Rc | 48.3 | 45.9 – 50.4 | 16.800 - 17.700 | 9.4 | | | 22.3 | -2.05 | |  |
|  |  | Rc/dark | 51.6 | 48.6 – 51.8 | 17.500 - 18.300 | 8.5 | | 22.5 | | | -0.11 |  |
| 5 | *Bc1* | Bc | 82.4 | 80.7 -83.7 | 25.700 - 26.200 | 8 | | 10.7 | | | -0.51 |  |
|  |  | FRc | 83.9 | 83.7 – 85.7 | 26.200 - 26.700 | 7.3 | | 16.3 | | | -1.01 |  |
|  |  | dark | 85.7 | 83.9 – 85.7 | 26.300 - 26.800 | 4.9 | | 11.2 | | | -1.05 |  |
| 5 | *FRc1* | FRc/dark | 2.6 | 1.95 – 2.8 | 200 - 600 | 6.5 | | 15.7 | | | +0.05 |  |
| 3 | *Bc2* | Bc | 76.7 | 75.7 – 78.4 | 21.000 - 21.600 | 5.1 | | 8.7 | | | -0.5 |  |
| 3 | *Dark1* | dark | 54.1 | 53.9 – 55 | 18.000 - 18.400 | 4.5 | | 10.4 | | | -1.03 |  |
| 4 | *Bc3* | Bc/dark | 27.3 | 25.5 – 28.7 | 17.100 - 17.700 | 4 | | 9.7 | | | +0.02 |  |
| 2 | *Rc2* | Rc/dark | 31.5 | 31.5 – 35.2 | 8.500 - 9.200 | 4 | | 6.8 | | | -0.07 |  |

**Suppl. Table 5: CAPS primers used for *Rc1* and *Bc1* QTLs segregation.**

| QTL | Target location in chromosome V | Restriction enzymes | Forward primer  (5' to 3') | Reverse primer  (5' to 3' on opposite strand) |
| --- | --- | --- | --- | --- |
| *Rc1* | 16060029 | EcoRI | TCAATCTCGCCGAAATCTCA | TGGTTGCTTTGAGTGGTGC |
|  | 16989903 | EcoRI | GGGCTTTCCGGTCTGATCT | TGACAACGACACTTACATGGC |
|  | 17922604 | EcoRI | TCGGAGAATGCTTTTGACGC | GAACAGAACCAAACCAAACC |
|  | 18989512 | EcoRI | GGGACTTCACGGGCGAAT | CCAACCGACTCCAGCACC |
| *Bc1* | 25089966 | EcoRI | TGCATACAGACCACAAGTG | CCAGTACTCACCACCGCC |
|  | 25801157 | EcoRI | TCACTCTTTCTCTCTGCCGC | GCCCTATATATACACCGAGGCG |
|  | 26382803 | EcoRI | CGGATGAAGAGTACACACGGT | TGAAGAGGGCTATCAGTGGC |
|  | 26796982 | XhoI | ACCGTCGAGAAACCATCCAC | CGATCCGAATACCCACAAAGAG |

**Suppl. Table 6: Annotated genes within each QTL interval according to Araport11.**

| **QTL** | **# genes per QTL** |
| --- | --- |
| *Rc1* | 458 |
| *Bc1* | 403 |
| *FRc1* | 150 |
| *Bc2* | 220 |
| *Dark1* | 131 |
| *Bc3* | 192 |
| *Rc2* | 249 |
| total | 1803 |

**Suppl. Table** **9:** **Candidate genes involved in de-etiolation occurring within each QTL.**

Light-related gen ontology (GO) candidates where assessed for occurrence of informative SNPs in Pat background, annotated as impactful polymorphisms according to SnpEff.

| **QTL** | **Gene** | | **Name** | **Missense variant** | | **Splice acceptor variant** | **Splice donor variant** | **Start lost** | **Stop gained** | **Stop lost** |
| --- | --- | --- | --- | --- | --- | --- | --- | --- | --- | --- |
| *Rc1* | *AT5G43890* | *YUC5* | | | 1 | 0 | 0 | 0 | 0 | 0 |
|  | *AT5G43470* | *RPP8* | | | 5 | 0 | 0 | 0 | 0 | 0 |
|  | *AT5G43630* | *TZP* | | | 6 | 0 | 0 | 0 | 1 | 0 |
|  | *AT5G43935* | *FLS6* | | | 9 | 0 | 0 | 0 | 0 | 0 |
|  | *AT5G44110* | *ABCI21* | | | 1 | 0 | 0 | 0 | 0 | 0 |
| *Bc1* | *AT5G64330* | *RPT3* | | | 1 | 0 | 0 | 0 | 0 | 0 |
|  | *AT5G67030* | *ABA1* | | | 5 | 0 | 0 | 0 | 0 | 0 |
|  | *AT5G66250* | kinectin-like protein | | | 3 | 0 | 0 | 0 | 0 | 0 |
| *FRc1* | *AT5G02200* | *FHL* | | | 2 | 0 | 0 | 0 | 0 | 0 |
| *Bc2* | *AT3G57180* | *BPG2* | | | 2 | 0 | 0 | 0 | 0 | 0 |
| *Bc3* | *AT4G37280* | *MRG1* | | | 1 | 0 | 0 | 0 | 0 | 0 |
|  | *AT4G36240* | *GATA7* | | | 1 | 0 | 0 | 0 | 0 | 0 |
| *Rc2* | *AT2G19690* | *PLA2-BETA* | | | 1 | 0 | 0 | 0 | 0 | 0 |

**Suppl. Table 10:** **Pat polymorphisms occurring in genes *TZP* and *ABA1*.**

Missense variants or stop codon gained allele coordinates marked as Col-0 reference (REF) or alternative (ALT) Pat within *TZP* and *ABA1* genes.

| Gene | Position | REF | ALT | Type | Transcript | Protein |
| --- | --- | --- | --- | --- | --- | --- |
| *TZP* | 17528075 | C | A | missense_variant | c.624C>A | p.Asp208Glu |
| *TZP* | 17528089 | G | A | missense_variant | c.638G>A | p.Gly213Asp |
| *TZP* | 17528730 | C | T | missense_variant | c.1279C>T | p.Pro427Ser |
| *TZP* | 17529288 | T | C | missense_variant | c.1837T>C | p.Cys613Arg |
| *TZP* | 17529477 | G | T | missense_variant | c.2026G>T | p.Ala676Ser |
| *TZP* | 17530127 | A | G | missense_variant | c.2416A>G | p.Arg806Gly |
| *TZP* | 17529973 | C | T | Stop_gained | c.2359C>T | p.Gln787* |
| *ABA1* | 26754495 | T | G | missense_variant | c.1638A>C | p.Glu546Asp |
| *ABA1* | 26754515 | C | T | missense_variant | c.1618G>A | p.Asp540Asn |
| *ABA1* | 26756598 | T | C | missense_variant | c.493A>G | p.Ile165Val |
| *ABA1* | 26756665 | T | A | missense_variant | c.426A>T | p.Glu142Asp |
| *ABA1* | 26756964 | G | A | missense_variant | c.127C>T | p.Pro43Ser |

**Suppl. Table 11: Accessions with the same informative Pat missense variants in candidate genes *TZP* and *ABA1*.**

Presence (1|1), absence (0|0) and missing data (./.) were called for each Pat informative coordinate across assayed accessions

| Gene | POS | Accessions | | | | | | |
| --- | --- | --- | --- | --- | --- | --- | --- | --- |
|  |  | Ting-1 | Tamm-2 | Tamm-27 | Sq-1 | Got-22 | Chat-1 | Ts-1 |
| *TZP* | 17528075 | 1\|1 | 1\|1 | 1\|1 | 1\|1 | 1\|1 | 1\|1 | 1\|1 |
| *TZP* | 17528089 | 1\|1 | 1\|1 | 1\|1 | 1\|1 | 1\|1 | 1\|1 | 1\|1 |
| *TZP* | 17528730 | 0\|0 | 0\|0 | 0\|0 | 0\|0 | 0\|0 | 0\|0 | 1\|1 |
| *TZP* | 17529288 | 0\|0 | 0\|0 | 0\|0 | 0\|0 | 0\|0 | 0\|0 | 1\|1 |
| *TZP* | 17529477 | 0\|0 | 0\|0 | 0\|0 | ./. | 0\|0 | 0\|0 | 1\|1 |
| *TZP* | 17530127 | 1\|1 | 1\|1 | 1\|1 | 1\|1 | 1\|1 | 1\|1 | 1\|1 |

| Gene | POS | Accessions | | | | |
| --- | --- | --- | --- | --- | --- | --- |
|  |  | RRS-10 | Ag-0 | Pna-17 | Dem-4 | Ts-1 |
| *ABA1* | 26754495 | 1\|1 | 1\|1 | 1\|1 | 1\|1 | 1\|1 |
| *ABA1* | 26754515 | 1\|1 | 1\|1 | 1\|1 | 1\|1 | 1\|1 |
| *ABA1* | 26756598 | 1\|1 | 1\|1 | 1\|1 | 1\|1 | 1\|1 |
| *ABA1* | 26756665 | 1\|1 | 1\|1 | 1\|1 | 1\|1 | 1\|1 |
| *ABA1* | 26756964 | 1\|1 | 1\|1 | 1\|1 | 1\|1 | 1\|1 |
